# Mite Infestation Induces a Moderate Oxidative Stress in Short-Term Soybean Exposure

**DOI:** 10.3390/plants14040590

**Published:** 2025-02-14

**Authors:** Wesley Borges Wurlitzer, Julia Renata Schneider, Joaquim A. G. Silveira, Maria Goreti de Almeida Oliveira, Noeli Juarez Ferla

**Affiliations:** 1 Laboratory of Acarology, Tecnovates, University of Vale do Taquari—Univates, Av. Avelino Talini, 171, Lajeado 95914-014, RS, Brazil; julia.schneider4@universo.univates.br; 2 Postgraduate Program in Biotechnology, University of Vale do Taquari—Univates, Av. Avelino Talini, 171, Lajeado 95914-014, RS, Brazil; 3 Plant Metabolism Laboratory (LabPlant), Department of Biochemistry and Molecular Biology, Federal, University of Ceará, Fortaleza 60451-970, CE, Brazil; silveira@ufc.br; 4 Bioagro-Instituto de Biotecnologia Aplicada à Agropecuária/INCT-Interações Planta-Praga, Universidade Federal de Viçosa—UFV, Viçosa 36570-900, MG, Brazil; malmeida@ufv.br

**Keywords:** biotic stress, plant physiology, redox metabolism, signaling, *Tetranychus ludeni*

## Abstract

Phytophagous mites are herbivores that feed on various economically important plants, such as soybean [*Glycine max* (L.) Merril]. Thus, our objective is to evaluate the oxidative stress stage of soybean plants infested by *Tetranychus ludeni* Zacher. Leaflets from three trifoliate leaves were pooled to form composite samples for each exposure time and evaluated at the following evaluation times: 0 h, 20 min, 6 h, 12 h, 24 h, and 48 h. In the initial phase of infestation (20 min), an oxidative burst was observed, represented by prominent hydrogen peroxide accumulation rather than superoxide radicals. This oxidative burst occurred in parallel to a strong increase in the antioxidant activities of catalase, ascorbate peroxidase, and glutathione *S*-transferase, but not in that of superoxide dismutase. These changes likely reflected an enhanced activation of signaling pathways involved in the oxidative stress response. After this initial phase, from 20 min to 6 h, a prominent decrease occurred in catalase, ascorbate peroxidase, and glutathione *S*-transferases activities, despite the hydrogen peroxide levels remaining significantly elevated, along with a marked but transient increase in the reduced glutathione content and proline. Interestingly, superoxide dismutase activity increased significantly after 6 h in parallel to lipid peroxidation, whereas the content of hydrogen peroxide remained elevated until 12 h of infestation. By the final evaluation, after 48 h of infestation, some redox indicators remained altered in relation to control plants, but in a state of moderate redox stress. Thus, in an unprecedented way, our data suggest that *T. ludeni* infestation triggered a moderate oxidative stress response in soybean plants. These findings highlight that proper monitoring and management can reduce economic losses without resorting to aggressive chemical interventions.

## 1. Introduction

Plants are challenged by severe threats resulting from climate change, including the increased frequency and intensity of abiotic (i.e., lack or excess of water, pH, and salinity) and biotic (i.e., pathogens and herbivorous arthropods) stresses, which lead to crop damage and significant productivity losses [1,2,3,4]. In soybean [*Glycine max* (L.) Merril] cultivation, for instance, regardless of the cropping system, *Mononychellus planki* (McGregor), *Tetranychus ludeni* Zacher, and *T. urticae* Koch (Acari: Tetranychidae) have been increasingly reported in recent years [5]. Plants and arthropods coexist and interact in numerous ways. One possible interaction is herbivory, considered deleterious, which involves a sophisticated system of herbivore perception and the activation of constitutive or induced defenses [6,7]. In this relationship, plant plasticity is directly related to their highly developed and refined sensory system, capable of rapidly activating defense and signaling mechanisms, allowing plants to perceive, memorize, and continuously learn [8,9].

It is well known that plant immunological recognition begins with Pattern Recognition Receptors (PRRs), which monitor the presence of biotic factors through different types of elicitors, such as herbivore-associated molecular patterns (HAMPs), pathogen-associated molecular patterns (PAMPS), and damage-associated molecular patterns (DAMPS) [10]. In these ways, herbivores are capable of inducing alterations in membrane potential (Vm) and cytosolic Ca^2+^ influxes and triggering the production of reactive nitrogen species (RNS) and reactive oxygen species (ROS) [11].

Reactive oxygen species (ROS) are produced by various organelles, including apoplasts, mitochondria, peroxisomes, and others [12]. These molecules function as extremely rapid and efficient signaling agents. Specifically, hydrogen peroxide (H_2_O_2_) can quickly traverse cells via aquaporins and peroxiporins [13]. ROS levels are generally regulated and maintained within appropriate ranges by the enzymatic defense machinery, particularly ascorbate peroxidase (APX), catalase (CAT), and superoxide dismutase (SOD) [12]. However, plants under biotic stress often exhibit uncoordinated and highly complex redox responses [14]. This complexity arises because herbivores, in addition to causing mechanical injuries, often secrete chemical compounds, including elicitors that induce defenses and effectors that suppress or modulate them [11,15,16]. For instance, *Bemisia tabaci* (Genn.) suppresses H_2_O_2_-mediated signaling processes in tomato *Solanum lycopersicum* L. plants via a ferritin effector (*BtFer1*) [17]. Similarly, *T. ludeni* infestation reduces H_2_O_2_ content in soybean plants, a process closely linked to elevated APX activity [18]. In fact, plants under biotic stress exhibit APX as an important peroxidase, responsible for converting H_2_O_2_ into H_2_O or O_2_ [16].

It is well known that ROS, when their homeostatic balance with antioxidants is disrupted, can lead to oxidative stress and, consequently, irreversible damage. In fact, ROS levels fluctuate over time, along with enzymatic and non-enzymatic defenses [19], characterizing different redox or homeostatic states. Thus, when analyzed from a spatiotemporal perspective over short periods, these fluctuations can provide a sophisticated indication of the oxidative stress stage in which plants find themselves [9]. That is, after the transient state in homeostasis triggered by environmental disturbances, plants may exhibit the following three stress stages: I—moderate stress, with no impact on essential functions; II—chronic stress, where the disturbance may collapse the plant and lead to death; and III—eustress, involving memory activation and resistance via epigenetic mechanisms in response to mild stresses [9]. To date, most studies have sought to understand disturbances in redox homeostasis over long infestation periods [14,18]. While these time intervals may be appropriate for the proposed objectives, they are evidently not effective in capturing the rapid signaling processes that precede the activation of defense mechanisms immediately after and throughout the infestation period. As a result, they do not accurately reflect the actual oxidative stress stage that the plants are in, nor do they indicate possible modulations caused by infestations.

In fact, it is well understood that redox metabolism plays a central role in plant–environment interactions, including signaling cascades of varying intensities, both locally, where the herbivore causes injury, and systemically, in other regions/modules that have not been attacked [20]. Additionally, ROS are involved in processes such as stress response, defense, recovery, memory, and tolerance [9]. Thus, our hypothesis is that biochemical indicators related to the redox metabolism, evaluated in a time-course with short time intervals, may be an appropriate methodology for inferring the oxidative stress stage of soybean plants infested by *T. ludeni*. We anticipate that during the infestation (A) ROS levels will peak upstream, acting as important signaling molecules; (B) antioxidant defense components (both enzymatic and non-enzymatic) will neutralize ROS; and finally, (C) the infestation will result in low levels of lipid peroxidation, indicating a state of moderate redox stress.

## 2. Results

### 2.1. Tetranychus ludeni Triggers an Oxidative Burst in Soybean Plants at the Onset of Infestation

**Figure 1:**
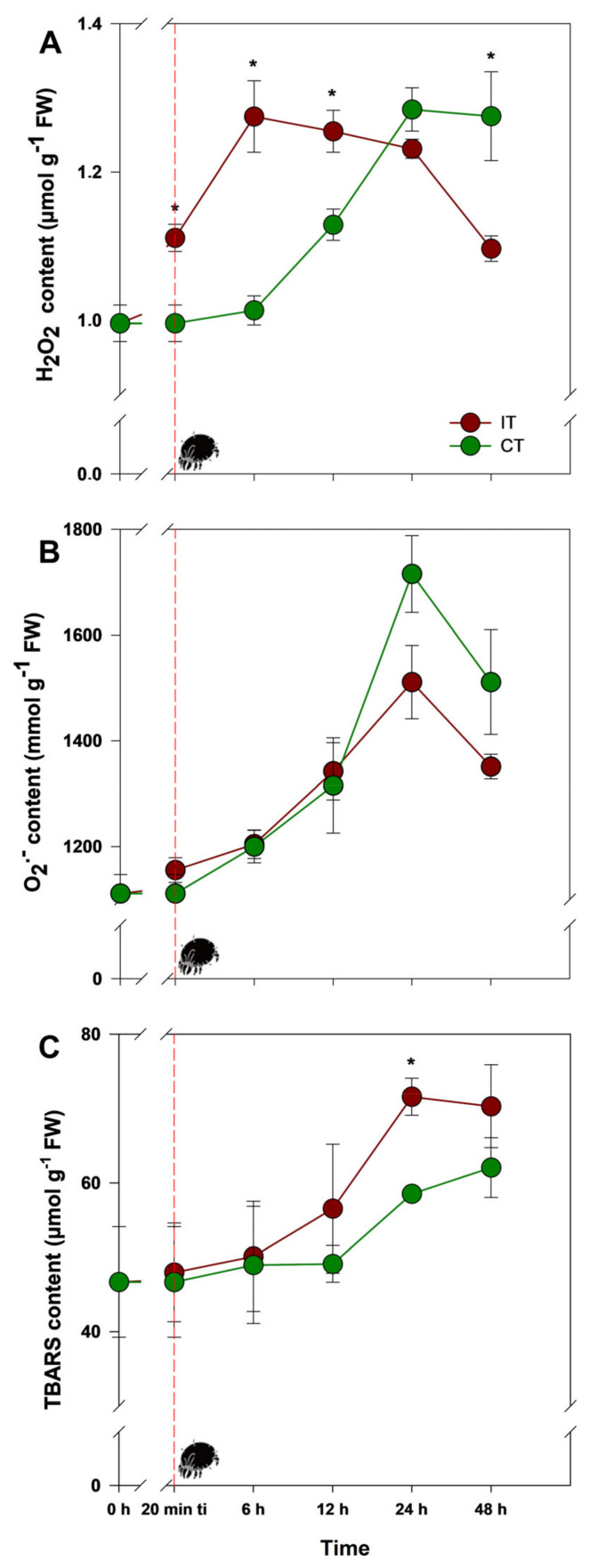
Content of (**A**) H_2_O_2_, (**B**) O_2_^•−^, and (**C**) TBARS (thiobarbituric acid reactive substances—lipid peroxidation) in soybean (*Glycine max*) plants infested by *Tetranychus ludeni* (IT—dark red) and control plants (CT—dark green) at the following evaluation times: 0 h, 20 min ti, 6 h, 12 h, 24 h, and 48 h. Asterisks indicate significant differences between treatments (IT and CT) (*t* test; *p* < 0.05). The vertical bars represent the ± standard error (*n* = 3). The dotted red line and the mite highlight the point 20 min after the initial phase of infestation (ti).

The infestation by *Tetranychus ludeni* in soybean plants triggered an intense and short-term signaling process, possibly mediated by hydrogen peroxide (H_2_O_2_). Indeed, in the plants infested by *T. ludeni* (IT), the increase in H_2_O_2_ levels began 20 min after the initial phase of infestation (ti), reaching a peak 6 h after ti (25% higher than control plants (CT)). On the other hand, 48 h after ti, unlike in CT plants, H_2_O_2_ levels consistently decreased in IT plants (13% lower than CT plants) (Figure 1A; Table S1). Regarding superoxide radicals (O_2_^•−^), although the levels were similar between treatments, a notable peak occurred 24 h after ti, beginning to decline after 48 h (Figure 1B; Table S1). In parallel, both IT and CT plants showed an increase in lipid peroxidation levels, especially 6 h after ti. However, a rapid accumulated oxidative effect, possibly caused by H_2_O_2_, resulted in a significant increase in lipid peroxidation indicated by TBARS (thiobarbituric acid reactive substances), particularly in IT plants, 24 h after ti (22% higher than CT plants), returning to levels close to CT plants 48 h after ti (Figure 1C; Table S1).

**Figure 2:**
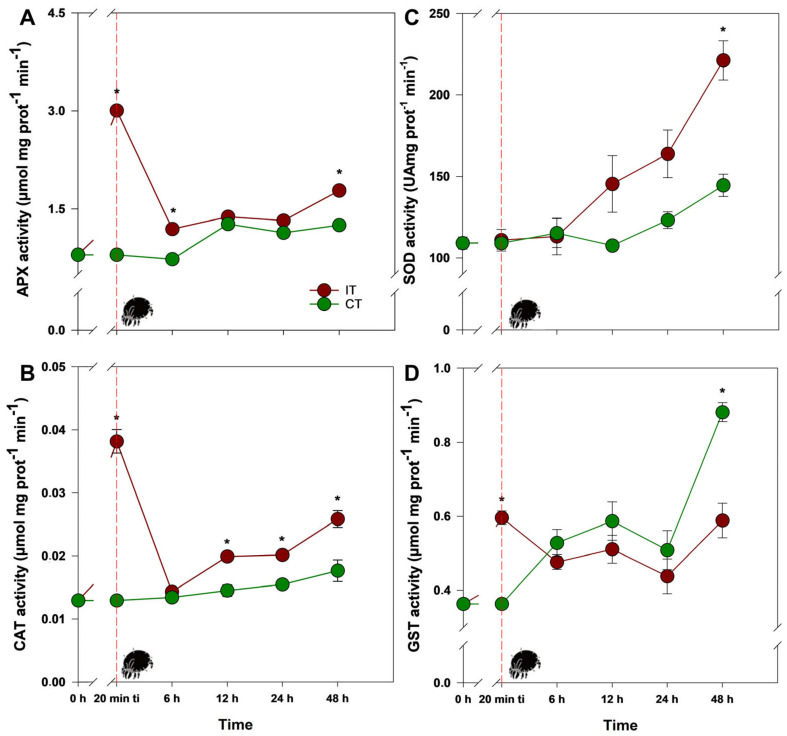
Activities of (**A**) APX, (**B**) CAT, (**C**) SOD, and (**D**) GST in soybean (*Glycine max*) plants infested by *Tetranychus ludeni* (IT—dark red) and control plants (CT—dark green) at the following evaluation times: 0 h, 20 min ti, 6 h, 12 h, 24 h, and 48 h. Asterisks indicate significant differences between treatments (IT and CT) (*t* test; *p* < 0.05). The vertical bars represent the ± standard error (*n* = 3). The dotted red line and the mite highlight the point 20 min after the initial phase of infestation (ti).

The rapid oxidative burst observed in IT plants was evidently associated with regulation in enzymatic and non-enzymatic antioxidant defense components. This was demonstrated by the activity of ascorbate peroxidase (APX) and catalase (CAT), which increased significantly at 20 min ti, by 277% and 195%, respectively, compared to CT plants, followed by a sharp decline (Figure 2A,B; Table S1). In other words, although APX activity remained higher in IT plants 6 h after ti, similar levels to CT plants were reached 12 h and 24 h after ti, returning to an increase 48 h after ti by 42% compared to CT plants. Similarly, CAT reached levels comparable to CT plants as early as 6 h after ti, increasing again 12 h after ti by 40% compared to CT plants, and remained stable with small fluctuations until the end of the experiment. Finally, it can be stated that oxidative and antioxidant responses are triggered in the first minutes of *T. ludeni* attack, progressively decline, and eventually reacquire a new redox state close to the initial one, which can be classified as an altered but reversible homeostatic state.

The activity of other important enzymes, such as superoxide dismutase (SOD) and glutathione *S*-transferase (GST), was also evaluated, and they showed fluctuations different from those observed in APX and CAT in IT plants. For example, SOD was stimulated in IT plants, especially 48 h after ti, reaching levels 43% higher compared to CT plants (Figure 2C; Table S1). Thus, considering the later stimulation of SOD activity, corroborated by adequate levels of O_2_^•−^ concentration, it is evident that *T. ludeni* infestation was not able to cause intense disturbances in this homeostasis. Regarding GST activity, IT plants showed a significant increase at 20 min ti, 64% higher compared to CT plants (Figure 2D; Table S1), gradually decreasing until reaching similar levels to CT plants 6 h after ti. These levels remained stable until 24 h after ti, at which point, interestingly, GST activity in CT plants significantly increased compared to IT plants, peaking 48 h after ti.

**Figure 3:**
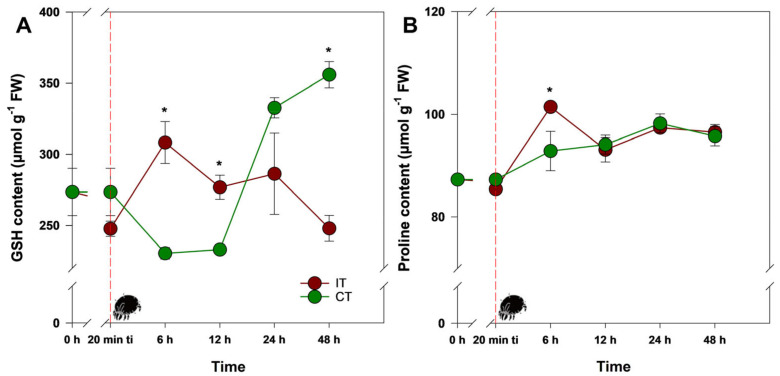
Content of (**A**) GSH and (**B**) proline in soybean (*Glycine max*) plants infested by *Tetranychus ludeni* (IT—dark red) and control plants (CT—dark green) at the following evaluation times: 0 h, 20 min ti, 6 h, 12 h, 24 h, and 48 h. Asterisks indicate significant differences between treatments (IT and CT) (*t* test; *p* < 0.05). The vertical bars represent the ± standard error (*n* = 3). The dotted red line and the mite highlight the point 20 min after the initial phase of infestation (ti).

As a non-enzymatic defense component necessary for GST activity, reduced glutathione (GSH) levels increased in IT plants, reaching values 34% higher than CT plants (Figure 3A; Table S1), particularly at 6 h after ti. Interestingly, 48 h after ti, while CT plants showed an increase in GSH levels, IT plants experienced a decrease in such levels, leading to a significant difference between treatments. This temporal reduction in GSH levels, coupled with the low GST activity, suggests that *T. ludeni* infestation in soybean plants triggers functional constraints between the substrate and the enzyme. Another key non-enzymatic defense component, proline, unexpectedly increased at 6 h after ti, being 12% higher in IT plants compared to CT plants (Figure 3B; Table S1). However, proline levels in IT plants returned to those of CT plants by 12 h after ti, indicating a rapid cell protective response to the intense signaling process induced by H_2_O_2_.

### 2.2. A General Perspective

**Figure 4:**
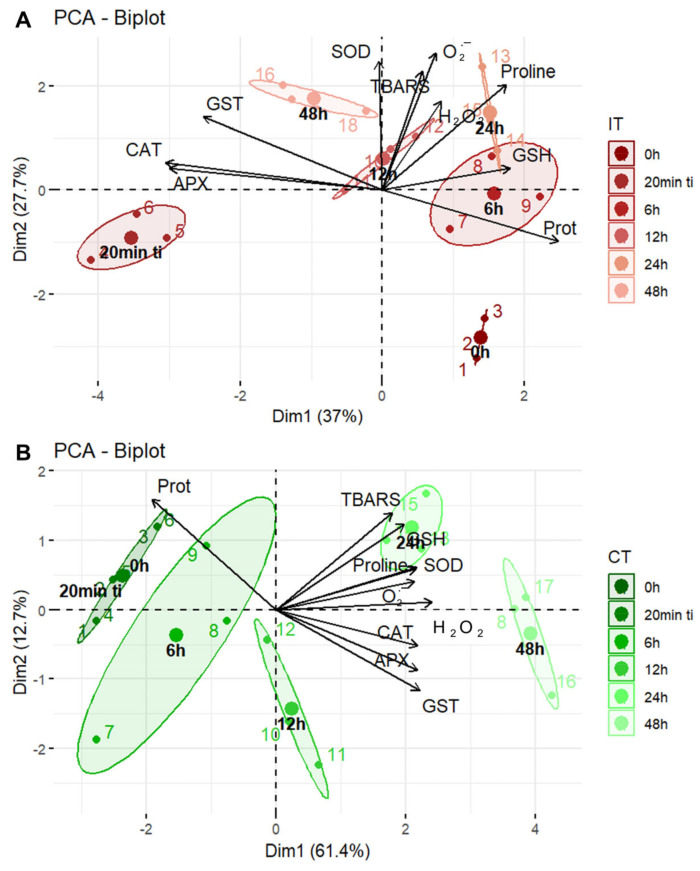
Principal Component Analysis (PCA) ordering of the evaluated variables in soybean (*Glycine max*) plants infested by *Tetranychus ludeni*—IT (**A**) and control plants CT (**B**)—at the following evaluation times: 0 h, 20 min ti, 6 h, 12 h, 24 h, and 48 h. The evaluated variables include the following: soluble protein (Prot), non-enzymatic antioxidants [proline and reduced glutathione (GSH)], antioxidant enzymes [glutathione *S*-transferase (GST), catalase (CAT), ascorbate peroxidase (APX) and superoxide dismutase (SOD)], reactive oxygen species [hydrogen peroxide (H_2_O_2_) and superoxide (O_2_^•−^)], and lipid peroxidation [thiobarbituric acid reactive substances (TBARS)].

The Principal Component Analysis (PCA) explained 64.7% (Figure 4A and Figure S1) and 74.1% (Figure 4B and Figure S2) of the total variance. In fact, although the PCA explained a substantial portion of the variance, the residual variance may contain additional biological variation not captured by the first two principal components. However, this residual variance does not diminish the patterns observed in the graphs. For IT plants, the variance explained by Principal Component 1 (PC1/Dim1) was 37%, and by Principal Component 2 (PC2/Dim2), 27.7%, with variations primarily associated with the activity of the enzymes CAT, APX, and GST, as well as the content of proline, soluble protein, and O_2_^•−^. For CT plants, PC1/Dim1 explained 61.4% of the variance and PC2/Dim2, 12.7%, with variations mainly related to the activity of GST and APX, protein content, and H_2_O_2_. Considering these key variables, IT plants showed distinct responses at different evaluation times after infestation. At 20 min ti, the responses were related to the activity of APX and CAT. At 6 h, the responses were associated with GSH content. At 12 h, the responses were linked to TBARS, H_2_O_2,_ and O_2_^•−^ levels. At 24 h, the responses involved proline content. At 48 h, the responses were dominated by SOD activity. For CT plants, most variables—particularly at 24 h after ti, were associated with TBARS, GSH, proline, SOD, and O_2_^•−^. These findings strongly support that *T. ludeni* infestation induces an intense and rapid oxidative activity closely tied to antioxidant responses.

**Figure 5:**
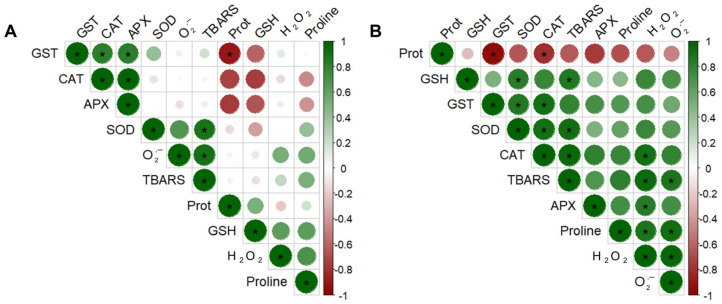
Pearson correlations among the variables assessed in soybean (*Glycine max*) plants infested by *Tetranychus ludeni*—IT (**A**) and control plants CT (**B**)—considering all evaluation times. The variables include soluble protein (Prot), non-enzymatic antioxidants [proline and reduced glutathione (GSH)], enzymatic antioxidants [glutathione *S*-transferase (GST), catalase (CAT), ascorbate peroxidase (APX) and superoxide dismutase (SOD)], reactive oxygen species [hydrogen peroxide (H_2_O_2_) and superoxide (O_2_)], and lipid peroxidation [thiobarbituric acid reactive substances (TBARS)]. Green and red circles with an asterisk represent positive and negative correlations (*p* < 0.05).

To provide a comprehensive understanding of the relationships between biochemical indicators of the redox metabolism and to reveal how homeostasis tends to adjust, a Pearson correlation analysis was conducted considering all evaluation times combined, capturing both positive and negative associations within each treatment (Figure 5A,B; Table S2). This analysis revealed a neutral scenario in IT plants, strongly indicating a balanced and adaptive response. Positive correlations were particularly evident between GST and CAT with APX and SOD and O_2_^•−^ with lipid peroxidation (Figure 5A; Table S2). Conversely, in CT plants, a predominance of positive correlations was observed, likely reflecting natural oscillations, which were especially pronounced between GST activity and other defense enzymes such as SOD and CAT, as well as among SOD, CAT, APX, and proline with ROS and lipid peroxidation (Figure 5B; Table S2).

**Figure 6:**
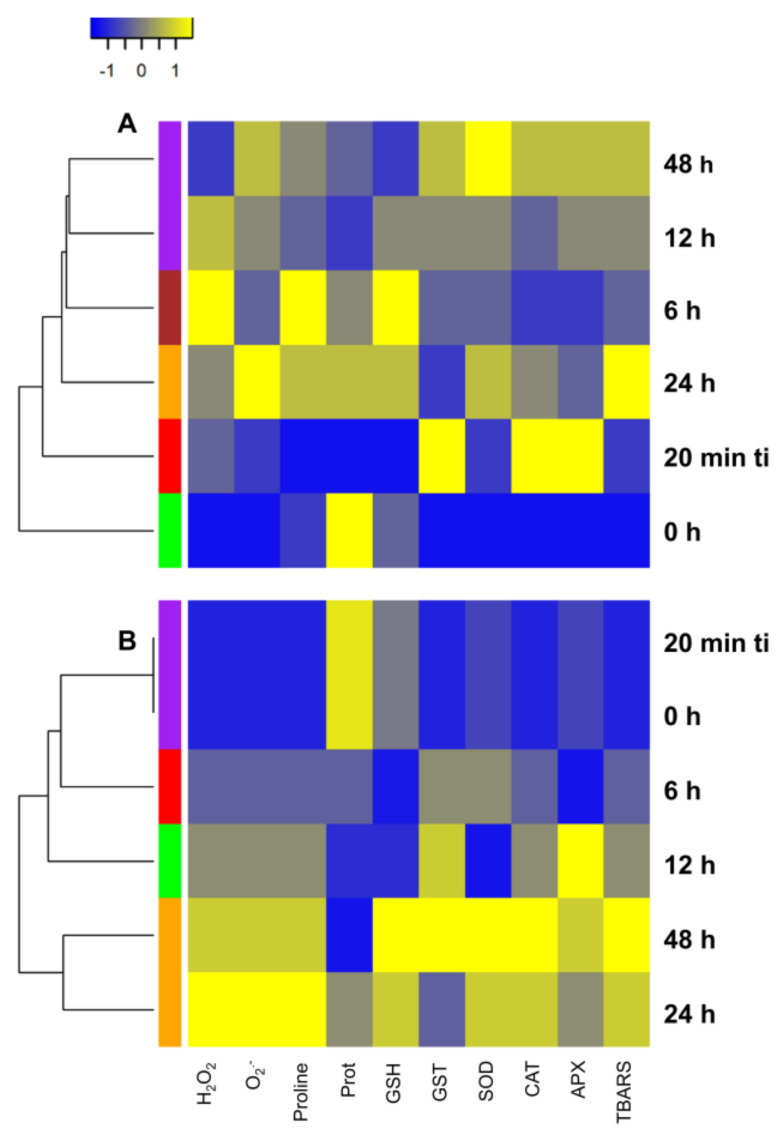
Heat map analysis of different evaluation times in relation to redox responses in soybean (*Glycine max*) plants infested by *Tetranychus ludeni*—IT (**A**) and control plants CT (**B**). The colors (green, red, brown, orange, and purple) in the Grouping Column indicate the different evaluation times (0 h, 20 min ti, 6 h, 12 h, 24 h, and 48 h) and identify four subclusters in CT and IT plants, along with the lines in each dendrogram. Redox responses include soluble protein (Prot), non-enzymatic antioxidants [proline and reduced glutathione (GSH)], enzymatic antioxidants [glutathione *S*-transferase (GST), catalase (CAT), ascorbate peroxidase (APX), and superoxide dismutase (SOD)], reactive oxygen species [hydrogen peroxide (H₂O₂) and superoxide (O_₂_^•−^)], and lipid peroxidation [thiobarbituric acid reactive substances (TBARS)]. The color gradient indicates the relative intensity levels of the variables, with more intense colors (yellow) representing higher intensity and cooler colors (blue) indicating lower intensity. The averages of the three biological replicates were considered.

In the heat map, the four main subclusters indicated by the Grouping Column, along with the dendrogram, corresponded to different evaluation times of redox responses in both IT and CT plants (Figure 6A,B). Thus, in IT plants, the dendrogram structure revealed similarities between redox responses at 48 h and 12 h after ti. An isolated cluster was formed at 0 h, indicating that before the onset of infestation, the plants’ redox responses were distinct from those at other evaluation times, especially from those at 48 h and 12 h after ti. Interestingly, in CT plants, the dendrogram structure differed completely from that of IT plants. The dendrogram revealed strong similarities, particularly between redox responses at 20 min ti and 0 h, as well as between 48 h and 24 h. Additionally, redox responses at 6 h were more similar to the subcluster of redox responses at 20 min ti and 0 h. The subcluster formed by redox responses at 48 h and 24 h was the most distinct. These results align with those observed in the PCA analysis. For instance, in CT plants, 6 h is close to 20 min ti and 0 h, whereas in IT plants, 48 h is close to 12 h, and 0 h forms a distant and isolated ellipse from the others.

## 3. Discussion

### 3.1. Could Soybean Plants Tolerate Tetranychus ludeni Infestation?

Evidently, stress caused by herbivory extends beyond mechanical injury, as elicitors and effectors are responsible for triggering intense signaling and defense cascades, making the understanding of stress intensity caused by herbivores, such as phytophagous mites, highly complex and lacking information [14]. Addressing this urgency, we proposed to detect the oxidative stress stage experienced by young soybean plants infested by *T. ludeni* through a time-course analysis of redox homeostasis. Our results revealed a state of moderate redox stress [9], characterized by an antioxidant burst of APX and CAT in the initial phase of infestation (ti) followed by a peak in H_2_O_2_ content. Although this led to an accumulated oxidative effect (lipid peroxidation), it ultimately resulted in a new stage, moderate oxidative stress, which highlighted an efficient defense and signaling process of the plant.

It is well known that tetranychid mites, when feeding, suck out the cellular content, especially from the active mesophyll cells [14]. In fact, important organelles are either sucked out or damaged, leading to the appearance of chlorotic lesions and a reduction in the photosynthetic rate in leaf tissue [14]. Although this study did not assess the long-term effects due to the short observation period, in prolonged infestations, photosynthetic indices tend to decrease and are associated with increased concentrations of ROS [18]. In this process, the accumulation of ROS occurs rapidly through the reduction of O_2_ by electrons generated mainly in chloroplasts, mitochondria, and peroxisomes [12]. Thus, the elevated levels of H_2_O_2_ observed in infested plants in this study, especially between 20 min ti and 12 h, are possibly, in part, a response to mechanical injuries. However, it is important to highlight that secretions injected during feeding may also contribute to this accumulation.

Although the literature lacks information, it is known that xenobiotics secreted by herbivores, such as elicitors and effectors, can trigger several unexpected responses in the signaling processes of their host plants [14,16]. That is, various peptides secreted in herbivore saliva can directly modulate the metabolic adjustments of host plants [10,11]. In this context, H_2_O_2_ has recently been recognized as a potential marker for responses related to elicitors, such as HAMPs, and effectors [10]. For example, in plant–mite interactions, tetranin (Tet2), a protein elicitor identified in the salivary glands of *T. urticae*, was capable of triggering ROS accumulation in beans (*Phaseoulus vulgaris* L.) [11]. Based on our results among the ROS evaluated, unusually, H_2_O_2_ was the first and only species accumulated in soybean plants infested by *T. ludeni*. *Tetranychus urticae* and *T. ludeni* are species within the same genus, sharing morphological characteristics indicative of strong similarity [21,22]. Thus, considering that H_2_O_2_ has been an effective signaler for responses associated with HAMPS [10], it is likely that *Tet2*-like proteins found in *T. urticae* are also present in the salivary glands of *T. ludeni*. This hypothesis could help to explain the results observed in this study; however, confirmation requires future investigations to identify and characterize the salivary proteins of *T. ludeni*, with a particular focus on their potential roles as elicitors.

From a homeostatic balance perspective, to infer oxidative stress stages in plants [9], we demonstrated that O_2_^•−^ was not accumulated in infested plants, whereas H_2_O_2_ showed a consistent increase up to 6 h after ti, followed by a gradual decrease after this time. Indeed, an increase in lipid peroxidation levels also occurred, particularly at 24 h after ti. Apparently, this relationship between H_2_O_2_ and lipid peroxidation is repeated in other studies involving the plants’ redox responses to phytophagous mite infestation [14]. More specifically, in the case of soybean plants infested with *T. ludeni* for 14 and 24 days, ROS accumulation and lipid peroxidation were not confirmed in the apical region of the plants [18]. Generally, lipid peroxidation is triggered initially by the formation of lipid alkyl radicals through hydrogen abstraction primarily by hydroxyl radicals (OH•) from polyunsaturated fatty acids (PUFAs), followed by the formation of conjugated dienes and peroxyl radicals that propagate lipid chain damage, and ultimately, radical termination, producing stable molecules such as malondialdehyde (MDA) [23,24]. For example, OH• is predominantly generated from H_2_O_2_ through the Fenton reaction [12]. Therefore, the rapid increase and subsequent gradual decline in H_2_O_2_ levels observed in our results strongly characterize a sufficient and robust antioxidant defense mechanism to cope with and mitigate oxidative stress induced by *T. ludeni*.

Oxidative enzymes such as SOD commonly represent the first line of defense against O_2_^•−^ in the apoplast, which is often produced by electrons donated from NADPH oxidase to molecular oxygen (O_2_) [13]. This essential mechanism of SOD can be inferred from our results, which indicated a homeostatic balance between SOD and O_2_^•−^ in infested plants. This suggests that soybean plants can preserve this redox balance even under infestation. As a result of this homeostasis, H_2_O_2_ production becomes evident due to the dismutation of O_2_^•−^ by SOD [12]. In this context, CAT, APX, and some GSTs, as well as GST-dependent peroxiredoxins, are crucial peroxidases that act simultaneously to dismutate H_2_O_2_ into H_2_O [25]. While CAT and APX are produced in various organelles, they are predominantly found in peroxisomes. Catalase eliminates large amounts of H_2_O_2_, whereas APX fine-tunes low H_2_O_2_ levels, enabling efficient signaling processes [25]. Based on our results, both enzymes exhibited an initial burst, indicating that H_2_O_2_ was indeed induced during the early moments of infestation but efficiently neutralized afterward. On the other hand, although GST activity showed an initial increase in infested plants, it subsequently remained relatively constant, suggesting that redox homeostasis demand was predominantly managed by CAT and APX. From this perspective, CAT and APX can also be considered potential targets for the development of plants more tolerant to these infestations.

A possible modulation by infestation cannot be ruled out. Among the few studies available in the literature, recent evidence has demonstrated that a GST (*GSTU43*) can regulate lignin biosynthesis in tomato plants, reducing stress caused by salinity [26]. It is well known that lignin acts as an effective physical defense barrier against both abiotic and biotic stresses, including mites [27]. Thus, the reduction in GST and GSH levels observed in our results could partially be explained by the ability of *T. ludeni* to manipulate genes related to this metabolic pathway, as suggested by Wurlitzer et al. [18]. This mechanism could benefit mites by facilitating the feeding process. Considering another important non-enzymatic stress-mitigating component, proline is an essential amino acid in plant cells that plays a role in protein biosynthesis, osmoprotection, and homeostatic regulation [28]. Specifically, in homeostatic maintenance, proline is known to effectively combat OH• [29]. In our results, for the first time in the literature, soybean plants infested with *T. ludeni* exhibited an increase in proline content alongside elevated H_2_O_2_ levels, strongly indicating the elimination of OH• through the Fenton reaction. Moreover, the fact that proline levels in infested plants quickly returned to those of control plants may help to explain the results of Wurlitzer et al. [18], which showed no accumulation of ROS or proline in the apical region of soybean plants evaluated at least 14 days after infestation. This highlights the plant’s ability to quickly reestablish homeostasis, even under infestation.

### 3.2. Who Regulates Whom?

Based on the scenario of our results, together with the contribution of the variables from the multivariate analysis, it was revealed that the main antioxidant enzymes were intensely orchestrated in the initial minutes of infestation in an attempt to achieve homeostatic adjustment in the infested plants, preventing more severe disturbances. This fact is clearly corroborated and complemented by the dendrogram of the heat map analysis, in which, shortly after the onset of infestation, the indicated redox responses still resembled the initial state, with visible changes especially at the end of the evaluations, which manifested a state of moderate redox stress. This new state characterized a stage of moderate oxidative stress, similar to that proposed by Silveira and Sousa [9], that was confirmed by the correlation analysis, which showed a state of neutrality among ROS, oxidative enzymes, and lipid peroxidation in the infested plants.

**Figure 7:**
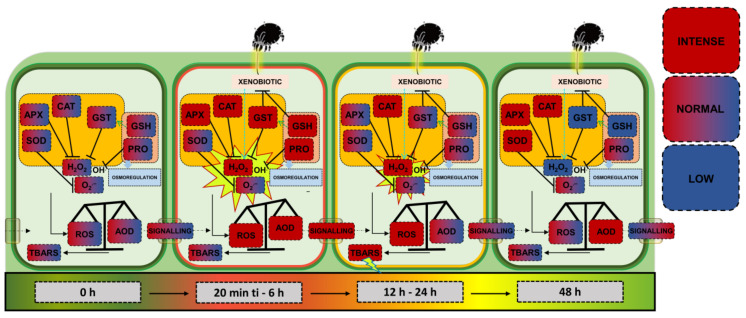
Schematic model to explain moderate oxidative stress in soybean (*Glycine max*) plants under infestation by *Tetranychus ludeni*. In a non-stressed state, plant cells without infestation exhibit biochemical indicators at a baseline intensity (0 h), configuring a fundamental redox state. Upon infestation, mechanical injuries and xenobiotic compounds (elicitors and effectors) secreted by the mites trigger an oxidative burst characterized by increased levels of oxidants and antioxidants, with elevated activities of CAT, APX, and GST neutralizing high levels of H_2_O_2_. As a result of this severe homeostatic disturbance, an intense apoplast-mediated signaling process occurs between cells (20 min ti–6 h). Subsequently, during a transitional yet less aggressive state, the accumulated oxidative effect, marked particularly by lipid peroxidation, is triggered. Moreover, although H_2_O_2_ is partially mitigated, CAT maintains an intense activity, tending to readjust homeostasis (12 h–24 h). Finally, despite the ongoing infestation, H_2_O_2_ levels consistently decrease. However, since GST and GSH are modulated by the infestation, APX resumes low-level activity, along with SOD, resulting in a new redox state similar to the initial one (state of moderate redox stress), reestablishing normal-intensity signaling processes (48 h). The side boxes indicate the intensity of biochemical indicators related to redox metabolism. The bar beneath the cells indicates the intensity of redox homeostasis oscillations: initial homeostasis—a non-stress scenario (0 h); oxidative burst—severe homeostatic disturbance (20 min ti to 6 h); reversible recovery—an intermediate homeostatic state between intense and weak stress (12 h to 24 h); and a new redox state—similar to the initial one (48 h).

From this perspective, numerous studies depict a scenario of an “arms race” in plant–herbivore interactions [30,31,32]. In this dynamic, while herbivores secrete elicitors and effectors, a cascade of signals, primarily mediated by ROS and hormones, is activated in host plants, triggering toxic or anti-nutritional properties against the predator [14,32]. Thus, the state of moderate redox stress observed here may not necessarily represent only an endogenous plant response. Instead, it could also reflect an adjustment induced by the mite to delay stress and keep the plant alive, ensuring food and shelter for an extended period (Figure 7).

## 4. Materials and Methods

### 4.1. Stock Colonies of T. ludeni

Stock colonies of *T*. *ludeni* were initiated from females collected from soybean fields located in the municipality of Muitos Capões (28°23′23″ S 51°15′12″ W), in the northern region of the state of Rio Grande do Sul, Brazil. The colonies were established and maintained in the laboratory since 2020, as previously described by Wurlitzer et al. [18]. The identification of *T. ludeni* was carried out using dichotomous keys [21,22] with the aid of a phase contrast optical microscope (Zeiss Imager Z2, Oberkochen, Germany).

### 4.2. Plant Growth and Treatment Conditions

For the establishment of the plants genotype FPS 2063 IPRO, expressing the *Cry* gene from the *Bacillus thuringiensis* var. kurstaki (Bt), soybean seeds were sown at a depth of 3–4 cm in 6 L plastic pots containing soil and substrate Carolina soil^®^ (pH 5.5; peat (turf), vermiculite, and limestone) in a 2:1 ratio. The seed treatment and the controlled conditions for plant growth were as described by Wurlitzer et al. [18]. Only the most vigorous plants were maintained [33].

### 4.3. Time-Course Experiment Design

The experiment was conducted using a total of 36 plants at the V3 growth stage [34]. Of these, 18 were subjected to infestation by *T. ludeni* (IT), while the remaining non-infested plants served as controls (CT). To take into account the plant globally and minimize the modular (spatial) effect, as demonstrated by Wurlitzer et al. [14,18], leaflets from the three trifoliate leaves were collected from three biological replicates (n = 3 plants), forming composite samples for the treatments (IT and CT) and their respective evaluation times: 0 h, 20 min, 6 h, 12 h, 24 h, and 48 h. The time for infestation took 20 min and started at 0 h (first sampling). Therefore, the 20 min time (second sampling) represents the initial phase of infestation (ti). The samples were carefully weighed and immediately frozen in liquid nitrogen (N_2_). The weight was recorded as fresh weight (FW), and the leaflets were ground in a mortar with a pestle under liquid nitrogen to obtain the extract. For plant infestation, 20 mobile forms of *T. ludeni* from the stock colony were transferred using a thin-tipped brush to each leaflet of the trifoliate leaves of each plant, totaling 180 mobile forms per plant. The experiment was conducted in a completely randomized design (CRD), comprising two treatments: IT and CT.

### 4.4. Obtaining Leaf Extract

The leaf extract was prepared for H_2_O_2_ reaction, and lipid peroxidation analysis followed the method of Loreto and Velikova [35], with slight modifications. Trichloroacetic acid (TCA) 0.1% was added to the plant powder in a ratio of 0.4:1.5 (*w*/*v*). The homogenate was then centrifuged at 2000× *g* for 20 min at 4 °C, and the supernatant (extract) was collected and stored at −80 °C for further analysis. The leaf extract preparation for evaluating proline content, reduced glutathione (GSH), antioxidant enzyme activity, O_2_^•−^ concentration, and total soluble protein concentration was adapted from the method used by Schneider et al. [36]. The plant powder was mixed with a standard buffer (170 mM sodium chloride, 3 mM potassium chloride, 5 mM dibasic sodium phosphate, and 2 mM monobasic sodium phosphate; pH 6.5) in a ratio of 1:4 (*w*/*v*). The homogenate was then centrifuged at 2000× *g* for 20 min at 4 °C, and the supernatant (extract) was collected and stored at −80 °C for subsequent analysis.

### 4.5. Levels of ROS and Lipid Peroxidation

The concentration of H_2_O_2_ was determined according to the methodology of Loreto and Velikova [35]. The analysis was conducted in triplicate, where 300 μL of extract was added to 10 mM potassium phosphate buffer (pH 7.0) and 1 M potassium iodide. The samples were kept in the dark for 15 min, and subsequently, absorbance was measured using a UV/VIS spectrophotometer (Shimadzu UV-1800, Kyoto, Japan) at 390 nm. The concentration of O_2_^•−^ was determined according to Chaitanya and Naithani [37]. The analysis was conducted in triplicate by adding 100 μL of the extract to a mixture containing 100 mM sodium phosphate buffer (pH 7.2), 1 mM sodium diethyldithiocarbamate, and 0.25 mM nitroblue tetrazolium. The absorbance of O_2_^•−^ was measured using a UV/VIS spectrophotometer (Shimadzu UV-1800) at 540 nm for 3 min with a time curve. The H_2_O_2_ content was expressed as μmol g^−1^ FW, and O_2_^•−^ content as mmol g^−1^ FW.

Lipid peroxidation was determined according to Heath and Packer [38], which quantifies the reactive substances present in the tissue associated with thiobarbituric acid (TBARS). To the extract was added 400 μL of 10% trichloroacetic acid and 0.67% thiobarbituric acid. The samples were incubated in a water bath at 100 °C for 15 min and then cooled and centrifuged at 1000× *g* for 15 min. The absorbance was measured using a UV/VIS spectrophotometer (Shimadzu UV-1800) at 535 nm, and the MDA content was calculated from the equation generated by the MDA standard curve. The TBARS content was expressed as μmol g^−1^ FW.

### 4.6. Antioxidant Enzyme Analysis

The activity of the enzyme ascorbate peroxidase (EC: 1.11.1.11) was quantified using the method of Nakano and Asada [39]. The analysis was performed in triplicate, and a reaction mixture was prepared with 50 mM potassium phosphate buffer (pH 6.8), 1 mM hydrogen peroxide, and 0.8 mM ascorbate. Enzymatic extract (50 μL) was added to the reaction mixture, homogenized, and the absorbance was read using a UV/VIS spectrophotometer (Shimadzu UV-1800) at 290 nm for 5 min, with a time curve, at 20 °C. The enzyme activity was calculated based on the ascorbate oxidation rate per min, considering an extinction coefficient of 2.8 mM^−1^ cm^−1^ [40]. The APX activity was expressed as µmol mg prot^−1^ min^−1^.

The enzyme catalase (EC: 1.11.1.6) was determined using the method of Cakmak et al. [41] with minor variations. This method evaluates the enzyme activity based on its ability to decompose H_2_O_2_. The analysis was performed in triplicate, and a reaction mixture was prepared with 50 mM potassium phosphate buffer (pH 6.8) and 20 mM H_2_O_2_. Enzymatic extract (50 μL) was homogenized with the reaction mixture (at 30 °C), and the absorbance was measured using a UV/VIS spectrophotometer (Shimadzu UV-1800) at 240 nm for 5 min, with a time curve, at 20 °C. The CAT activity was calculated according to the molar extinction coefficient of H_2_O_2_ (36,000 mM^−1^ cm^−1^) [40]. The CAT activity was expressed as µmol mg prot^−1^ min^−1^.

The activity of superoxide dismutase (EC: 1.15.1.1) was determined as proposed by Del Longo et al. [42], where one unit of activity (UA) of SOD was defined as the amount of enzyme required to inhibit the photoreduction of NBT by 50% [43]. The activity determination was performed in triplicate, using a reaction mixture prepared with 50 mM potassium phosphate buffer (pH 7.8), 13 mM methionine, 75 μM NBT, 0.1 mM ethylenediaminetetraacetic acid (EDTA), and 2 μM riboflavin. To this mixture was added 40 μL of enzyme extract. The samples were incubated at 25 °C and exposed to a 15 W lamp for ten min. After this period, a single reading was taken using a UV/VIS spectrophotometer (Shimadzu UV-1800) at 560 nm, at 20 °C. This reading allows for the determination of the formazan blue produced from the photoreduction of NBT [44]. The calculation of SOD activity took into account the difference between the readings of the samples exposed to light and those incubated in the dark [36]. SOD activity was expressed as UA mg prot^−1^ min^−1^.

The activity of the enzyme glutathione *S*-transferase (GST, EC: 2.5.1.13) was quantified using the methodology of Dalton [45]. The preparation involved adding 500 μL of the extract to the extraction buffer (200 mM potassium phosphate buffer, pH 7.8, 10 mM EDTA, and 5 mM ß-mercaptoetanol). Then, 100 μL of this extract was added to the incubation buffer (200 mM potassium phosphate buffer, pH 7.5, and 20 mM reduced glutathione) and 20 mM 1-chloro-2,4-dinitrobenzeno (CDNB), homogenized, and the absorbance was read in triplicate using a UV/VIS spectrophotometer (Shimadzu UV-1800) at 340 nm for 2 min, with a time curve, at 20 °C. The enzyme activity was analyzed by monitoring the formation of the conjugate between reduced glutathione and CDNB, which absorbs maximally at 340 nm with an extinction coefficient of 9.6 mM^−1^ cm^−1^ [46]. GST activity was expressed as µmol mg prot^−1^ min^−1^.

### 4.7. Non-Enzymatic Antioxidant Analysis

The determination of proline followed the principle of the original method proposed by Bates et al. [47] adapted from Shabnam et al. [48]. The previously prepared enzymatic extract was acidified with 3% sulfosalicylic acid. This extract was homogenized and pipetted into an acidic ninhydrin solution (0.0625 g of ninhydrin in 15 mL of glacial acetic acid), homogenized, and placed in a water bath at 100 °C for 30 min. After this period, the reaction was stopped in an ice bath, and the reading was taken at 508 nm using a UV/VIS spectrophotometer (Shimadzu UV-1800). The readings were performed in triplicate, and the proline concentration was estimated through a standard curve of proline. The proline content was expressed as µmol g^−1^ FW.

The content of GSH content was assayed as described by Griffith [49]. In Falcon tubes, 500 µL of enzyme extract was added, followed by the addition of 500 µL of 10% TCA. The tubes were centrifuged 250× *g* for 20 min. From each tube, 800 µL of the supernatant was taken and reacted with 80 µL of 5,5″-dithiobis-2-nitrobenzoic acid (DTNB). This mixture was transferred to a 1 mL cuvette for absorbance reading in a spectrophotometer (Shimadzu UV-1800) at 412 nm [36]. The content of GSH was subsequently calculated based on the equation obtained from the curve generated using cysteine standards. The GSH content was expressed as µmol g^−1^ FW.

### 4.8. Determination of Total Soluble Protein Concentration

The concentration of total soluble proteins in the crude leaf extracts was determined using the [50] method, with bovine serum albumin (BSA) as the standard protein. The analyses were performed using a UV/VIS spectrophotometer (Shimadzu UV-1800) at a wavelength of 595 nm, in triplicate.

### 4.9. Statistical Analysis

The data were analyzed using the Shapiro–Wilk test to assess the normality of residuals and the F-test for analysis of variance. Each treatment (CT and IT) was compared at each evaluation time using a *t*-test (*p* < 0.05; Table S1). All analyses were conducted in RStudio 4.3.2 [51]. Relationships between the variables in CT and IT were evaluated using Pearson correlation with the corrplot package [52]. A heat map was constructed using the clustering method with Canberra distance as a similarity measure and the Ward’s linkage method, with gplots RcolorBrewer and preprocess Core packages. Additionally, a multivariate analysis was performed using the principal component method with the FactoMineR [53] and factoextra [54] packages. Correlation, multivariate analysis, and heat map graphs were generated in RStudio [51], while line graphs with error bars, comparing CT and IT over time for each variable, were created using SigmaPlot 15.0 (Systat Software Inc., Richmond, CA, USA).

## 5. Conclusions and Perspectives

Considering redox homeostasis as an indicator of stress stages in plants is a complex challenge. In this study, we evaluated the time-course over a short term in young soybean plants, focusing particularly on oxidative stress. Unprecedentedly, we detected a moderate oxidative stress stage in soybean plants infested by *T. ludeni*. In the early minutes following infestation, an intense transitional state was evidenced by an oxidative burst, primarily triggered by the action of CAT and APX enzymes, alongside high levels of H_2_O_2_, promoting an aggressive signaling process. As a result of this signaling, an accumulated oxidative effect was observed, manifesting mainly through lipid peroxidation 24 h ti. Interestingly, even in the continuous presence of mites, H_2_O_2_ levels were consistently attenuated, and lipid peroxidation returned to normal levels 48 h after the onset of infestation. Furthermore, while GST and GSH levels decreased, APX activity resumed at low levels, along with SOD, leading to moderate redox stress by the absence of significant oxidative stress and a balanced signaling process. Based on these unprecedented discoveries, we can highlight the following two topics to be considered innovative for the area of plant physiology and management of *T. ludeni* in soybean crops:

- This study demonstrates that soybean plants under infestation by phytophagous mites exhibit a moderate redox stress or a new redox state characterized as a non-significant stressful oxidative. This condition reflects the plants’ intrinsic physiological ability to withstand infestation without significant damage. This finding is extremely relevant in the current agricultural context, as it opens the possibility of reducing the use of pesticides in soybean management. Thus, producers can adopt strategies based on cultural practices to control these phytophagous mites, promoting a more sustainable and cost-effective approach to agricultural production.
- Additionally, it was observed that antioxidant enzymes, particularly APX (ascorbate peroxidase) and CAT (catalase), displayed extremely rapid responses within the first minutes following infestation. Notably, this is the first study to demonstrate such immediate antioxidant defense mechanisms related to the redox metabolism of soybean plants under a biotic factor. These findings have significant potential to support biotechnological and breeding programs aimed at developing more tolerant cultivars. Exploring these rapid antioxidant responses may become a critical tool for enhancing plant tolerance to biotic factors, contributing to food security and the sustainability of modern agriculture.

Although this study has brought essential information on plant–pest interactions, further studies are needed, particularly those exploring longer infestation periods, which are strongly recommended. Such studies should encompass comprehensive evaluations of biomass levels, including both aerial parts and roots, as well as productivity indices, to provide a more in-depth understanding of plant responses. Additionally, research should investigate the capacity of mites to manipulate plant defenses, with a special focus on identifying and characterizing potential elicitor and effector secretions. To achieve these insights, the application of molecular tools is crucial, enabling detailed analyses of the biochemical and genetic pathways involved.

## Data Availability

Data are contained within the article.

## Supplementary Materials

The following supporting information can be downloaded at: https://www.mdpi.com/article/10.3390/plants14040590/s1, Figure S1: A. Scree plot with the percentage of explained variances. B. Contribution of the variables on the PCA of the infested plants (IT); Figure S2. A. Scree plot with the percentage of explained variances. B. Contribution of the variables on the PCA of the control plants (CT); Table S1: Summary T test and Table S2: Pearson Correlation.
