# Supplementary material for: Mite Infestation Induces a Moderate Oxidative Stress in Short-Term Soybean Exposure
Source: Plants (Basel). 2025 Feb 14;14(4):590. doi: 10.3390/plants14040590 (PMC11859938; doi:10.3390/plants14040590)
Supplement: Supplementary file 1 [file plants-14-00590-s001.zip › Figure S1 and S2.pdf]

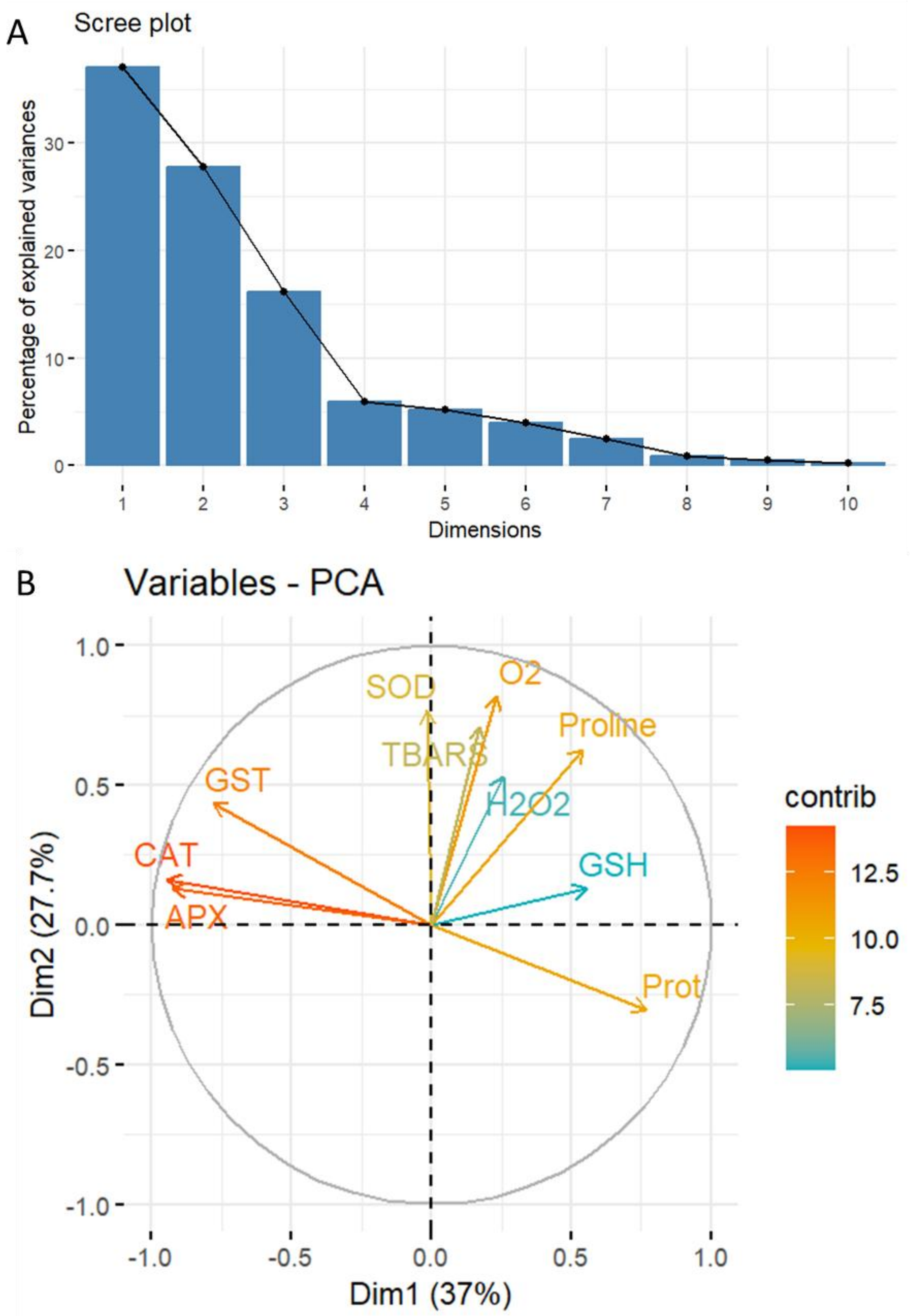

Figure S1. A. Scree plot with the percentage of explained variances. B. Contribution of the variables on the PCA of the infested plants (IT).

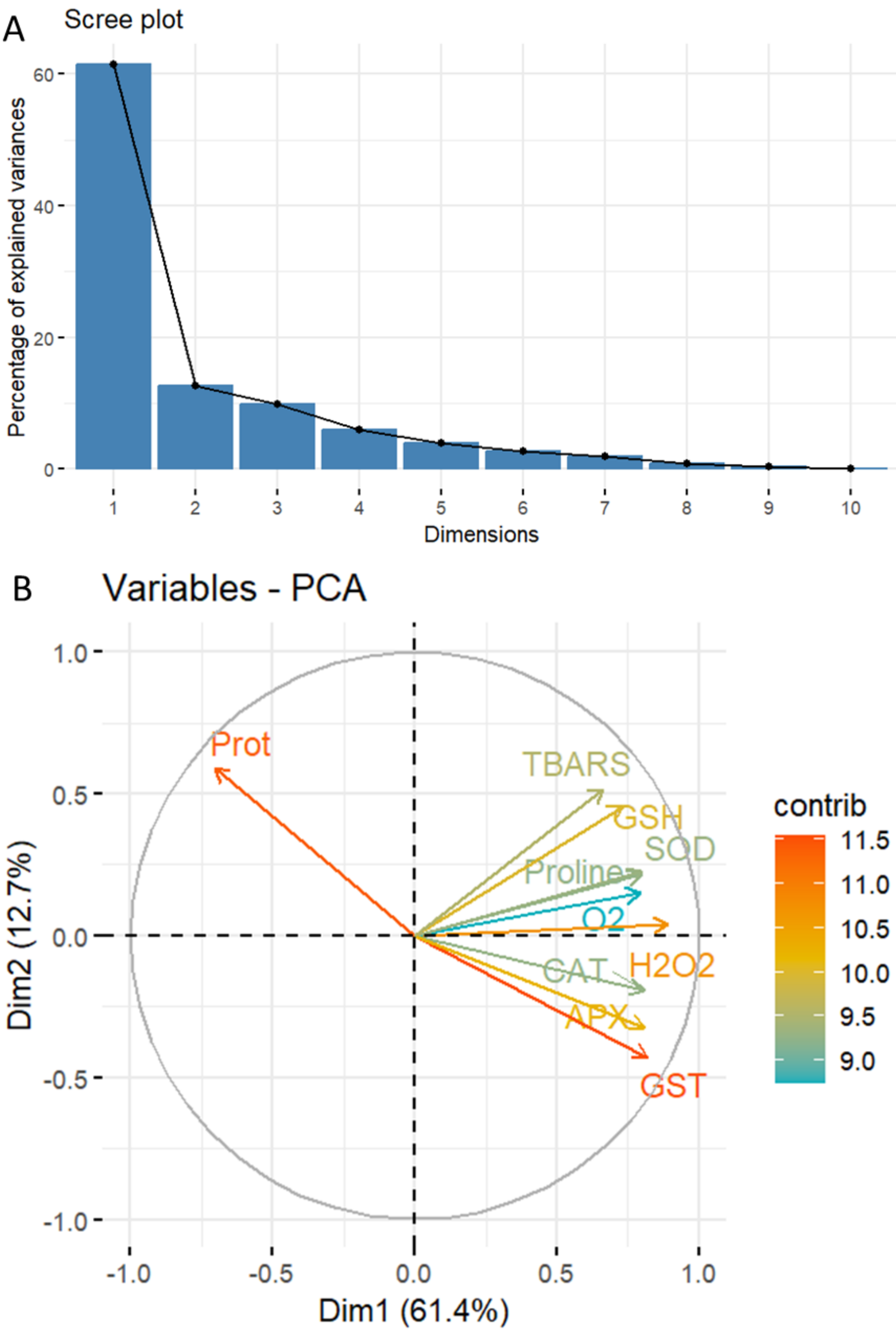

Figure S2. A. Scree plot with the percentage of explained variances. B. Contribution of the variables on the PCA of the control plants (CT).
